# Editorial: Autoantibodies in cancer: diagnostic, prognostic, and therapeutic potential

**DOI:** 10.3389/fonc.2026.1886097

**Published:** 2026-06-05

**Authors:** Cuiling Zheng

**Affiliations:** Department of Clinical Laboratory,National Cancer Center/National Clinical Research Center for Cancer/Cancer Hospital, Chinese Academy of Medical Sciences and Peking Union Medical College, Beijing, China

**Keywords:** clinical applications, detection methods, integrative perspective, tumor antigen-associated autoantibodies, underlying mechanisms

## 1 Introduction

More than a century ago, research indicated that the immune system could restrain the development of tumor, but tumor immunology is still considered a burgeoning field (1). Groundbreaking discoveries have recently clarified the activation of the body’s immune surveillance function since the early stage of tumor or, conversely, fostering the progression of tumor, which underlines the roles of distinct immune components in tumor development and their prognostic value (2–4). Even at the early tumor stage, the immune system is capable of triggering innate and adaptive immune cells to response against tumor-associated antigens (TAAs), which are a distinct group of cellular proteins in tumor patient plasma triggering spontaneous autoantibody reactions (5). This results in the presence of corresponding autoantibodies, which can also be detected in the plasma (6). Autoantibodies have been detected for a range of cancers at an early stage before development of clinical symptoms. This suggests that tumor-associated autoantibodies may serve as immunodiagnostic markers for the early diagnosis of tumor (7, 8). Tumor-associated autoantibodies (TAAbs) are stable and persist at high levels in the plasma of tumor patients for extended periods, especially in comparison to their absence in normal individuals and non-malignant conditions (2), which makes them attractive tools and becomes one of the most often tested biomarkers in the diagnosis and treatment of tumor patients. Conventional serum levels of TAAs, which are frequently used to track therapy effectiveness and evaluate prognosis, are challenging to identify in the early stages of tumor since they rise with tumor size (Tai and Zheng).

## 2 Detection methods of TAAbs

There are many established technologies that are minimally invasive, cost-effective, and easily performed to detect autoantibodies (9). To date, many autoantibody discovery technologies such as protein chip (Xiong et al.), immunofluorescence assay (Chen et al.) and enzyme-linked immunosorbent assay (ELISA) (Xiong et al.), have been used to detect tumor-associated autoantibodies. Protein chip technology facilitates high-throughput and simultaneous detection of hundreds of autoantibodies (10). Furthermore, its exceptional sensitivity allows for the identification of autoantibodies even at very low concentrations (11, 12). Immunofluorescence assay was performed to confirm the presence of tumor-associated autoantibodies such as proteinase 3 (PRTN3). While the American College of Rheumatology recommended the method of indirect immunofluorescence assay (IFA) using HEp-2 cells for autoantibodies (AAbs) screening, the interobserver variability in the result interpretation and different cutoff values in clinical applications greatly affected its clinical application (13).

## 3 Clinical applications of TAAbs

As for clinical applications, TAAbs have gained attention for their role as disease biomarkers (14). TAAbs demonstrate early emergence during carcinogenesis, offering potential as non-invasive diagnostic biomarkers for cancer diagnosis and distinguishing between benign and malignant nodules (Xiong et al., 15–17). TAAbs offer several advantages for early diagnosis in tumor: they can be detected years prior to clinical manifestation, exhibit remarkable stability in serum for up to 3 months, and demonstrate higher titers than their target antigens, enabling easy detection by Enzyme-linked immunosorbent assay (ELISA) (18–20). For example, cancer patients exhibit increased ANA, aPL, and ATA frequencies compared to healthy individuals (Chen et al.). The anti-OLA1 autoantibody shows its diagnostic value in HCC, especially in AFP-negative patients (Xiong et al.). Furthermore, TAAbs may demonstrates their prognostic value for non-small-cell lung cancer patients initiating immuno-therapy, with their occurrence associated with favorable prognosis (21).

## 4 Underlying mechanisms of TAAbs

Although much attention has been gained for the role of TAAbs as disease biomarkers, the underlying mechanisms remain incompletely understood. TAAbs have been regarded as a crucial bridge linking autoimmunity and malignancy. Tumors develop when the immune system’s surveillance role fails, permitting tumor antigens to evade detection, elimination, or immune evasion in multiple ways (Tai and Zheng ​). The creation and exposure to TAAs provoke the activation and proliferation of B lymphocytes, resulting in the production of TAAbs. Concurrently, antigen-presenting cells (including macrophages and dendritic cells) can identify, seize, process, and display them to T lymphocytes. This step stimulates the secretion of cytokines and chemokines, establishing a cascade that enhances autoantibody production (Tai and Zheng ​). The production of TAAbs offers direct evidence of active immune responses during neoplasia. However, according to the current literature, Tai and Zheng ​hypothesizes that the underlying mechanisms of TAAbs generation include, but are not limited to, the following: (i) protein overexpression, aberrant expression, and structural modifications; (ii) deficiencies in immune tolerance; (iii) the inflammatory tumor microenvironment; (iv) exosomes-mediated effects and immunogenic cell death; (v) non-classical immunoglobulins abnormally expressed by tumor cells. These autoantibodies not only reflect the dysregulated state of the immune system but also actively create tumour-promoting microenvironments by disrupting key signaling pathways, mimicking antigenic stimulation, and forming immune complexes (22). BCR/TLR7 signaling promotes autoantibody-producing plasma cell differentiation, which correlates with antitumor responses and may explain the frequent association between immunotherapy efficacy and autoimmune toxicity (23, 24). Moreover, inflammatory cytokines (TNF-a, IL-1, IL-6) at tumor sites create a pro-autoimmune milieu where the immune system generates TAAbs, potentially increasing autoimmune risk (25).

## 5 Integrative perspective of TAAbs

In recent years, many significant advances have been made in the research on autoantibodies and their combinations in tumors, but numerous challenges remain. First, future investigations are needed to find out the specific roles of serum TAAbs in tumor development, progression, and immunotherapy response across various tumor types. Second, future research should prioritise the development of risk prediction models informed by the combinations of TAAbs in tumors to enable early identification and precision intervention in high-risk individuals. Third, establishing large prospective cohorts and real-world study platforms for long-term monitoring of patients’ immune repertoires and clonal evolution trajectories will facilitate the discovery of diagnostic and predictive biomarkers. Finally, TAAbs not only reflect the dysregulated state of the immune system but also actively create tumour-promoting microenvironments by disrupting key signalling pathways, mimicking antigenic stimulation, and forming immune complexes (22). Therefore, the interaction between TAAbs and the tumor microenvironment is crucial for elucidating the molecular and cellular events driving malignant transformation and more attention should be paid to through chronic inflammation and extensive cell death.

## 6 Conclusions

Overall, our Research Topic highlights the roles of autoantibodies and the autoreactivity of immunocytes in tumor related autoimmunity. The critical roles of TAAbs in tumor pathogenesis and progression are not fully comprehended, as preclinical and clinical data are still limited. Nevertheless, TAAbs are remarkably persistent proteins that can be detected months to years prior to the onset of clinical signs in tumor patients, remaining present long after tumor antigens have vanished (26, 27). This characteristic establishes TAAbs as prospective biomarkers for early tumor diagnosis, prognostic evaluation, and therapy efficacy assessment. A deeper understanding of TAAbs could help us clarify the intersection of lymphoid structures’ biology, immune tolerance, and cancer immunity. Resolving how localized immune responses within the tumor microenvironment contribute to effective tumor resolution may uncover novel biomarkers and therapeutic targets. The findings of autoantibodies and their combinations in tumor not only holds the promise of enhancing biomarker research but also offers new therapeutic strategies for tumor immunoprevention.
